# Yeast-expressed recombinant As16 protects mice against *Ascaris suum* infection through induction of a Th2-skewed immune response

**DOI:** 10.1371/journal.pntd.0005769

**Published:** 2017-07-14

**Authors:** Junfei Wei, Leroy Versteeg, Zhuyun Liu, Brian Keegan, Ana Clara Gazzinelli-Guimarães, Ricardo T. Fujiwara, Neima Briggs, Kathryn M. Jones, Ulrich Strych, Coreen M. Beaumier, Maria Elena Bottazzi, Peter J. Hotez, Bin Zhan

**Affiliations:** 1 Texas Children’s Hospital Center for Vaccine Development, National School of Tropical Medicine, Baylor College of Medicine, Houston, Texas, United States of America; 2 Departamento de Parasitologia, Universidade Federal de Minas Gerais, Belo Horizonte, Brazil; 3 The University of Texas MD Anderson Cancer Center UT Health Graduate School of Biomedical Sciences, Houston, Texas, United States of America; 4 Department of Biology, Baylor University, Waco, Texas, United States of America; Queen's University Belfast, UNITED KINGDOM

## Abstract

**Background:**

*Ascariasis* remains the most common helminth infection in humans. As an alternative or complementary approach to global deworming, a pan-anthelminthic vaccine is under development targeting *Ascaris*, hookworm, and *Trichuris* infections. As16 and As14 have previously been described as two genetically related proteins from *Ascaris suum* that induced protective immunity in mice when formulated with cholera toxin B subunit (CTB) as an adjuvant, but the exact protective mechanism was not well understood.

**Methodology/Principal findings:**

As16 and As14 were highly expressed as soluble recombinant proteins (rAs16 and rAs14) in *Pichia pastoris*. The yeast-expressed rAs16 was highly recognized by immune sera from mice infected with *A*. *suum* eggs and elicited 99.6% protection against *A*. *suum* re-infection. Mice immunized with rAs16 formulated with ISA720 displayed significant larva reduction (36.7%) and stunted larval development against *A*. *suum* eggs challenge. The protective immunity was associated with a predominant Th2-type response characterized by high titers of serological IgG1 (IgG1/IgG2a > 2000) and high levels of IL-4 and IL-5 produced by restimulated splenocytes. A similar level of protection was observed in mice immunized with rAs16 formulated with alum (Alhydrogel), known to induce mainly a Th2-type immune response, whereas mice immunized with rAs16 formulated with MPLA or AddaVax, both known to induce a Th1-type biased response, were not significantly protected against *A*. *suum* infection. The rAs14 protein was not recognized by *A*. *suum* infected mouse sera and mice immunized with rAs14 formulated with ISA720 did not show significant protection against challenge infection, possibly due to the protein’s inaccessibility to the host immune system or a Th1-type response was induced which would counter a protective Th2-type response.

**Conclusions/Significance:**

Yeast-expressed rAs16 formulated with ISA720 or alum induced significant protection in mice against *A*. *suum* egg challenge that associates with a Th2-skewed immune response, suggesting that rAS16 could be a feasible vaccine candidate against ascariasis.

## Author summary

Roundworms (*Ascaris*) infect more than 700 million people living in poverty worldwide and cause malnutrition and physical and mental developmental delays in children. As an alternative or complementary approach to global deworming, a pan-anthelminthic vaccine is under development that targets ascariasis in addition to other human intestinal nematode infections. Towards this goal, two *Ascaris suum* antigens, As16 and As14, were expressed in *Pichia pastoris* as recombinant proteins. Mice immunized with rAs16 formulated with ISA720 adjuvant produced significant larva reduction (36.7%) and stunted larval development against *A*. *suum* egg challenge. The protection was associated with predominant Th2-type responses characterized by high levels of serological IgG1 (IgG1/IgG2a > 2,000) and Th2 cytokines, IL-4 and IL-5. A similar level of protection was observed in mice immunized with rAs16 formulated with alum that induces mainly a Th2-type immune response, whereas mice immunized with rAs16 formulated with MPLA or AddaVax, both inducing major Th1-type responses, were not significantly protected against *A*. *suum* infection. High-yield expression of rAs16 in yeast will allow for large-scale manufacture, and its protective efficacy when formulated with alum suggests its suitability as a vaccine candidate.

## Introduction

*Ascaris lumbricoides*, *Trichuris trichiura* and the hookworm *Necator americanus* are the three major soil-transmitted helminths (STH) that infect more than one billion poor people in the world and are the leading neglected tropical diseases (NTDs) in terms of disability-adjusted life years (DALYs) [1]. New estimates from the Global Burden of Disease Study 2015 indicate that approximately 761 million people are chronically infected with *A*. *lumbricoides*, resulting in 2,700 annual deaths from ascariasis [2, 3]. Global control of STH infections depends on the mass drug administration of anthelminthics such as albendazole or mebendazole targeting children between the ages of 1–14 years [4]. However, the rapid rates of post-treatment re-infection [5], potential drug resistance [6], low treatment coverage for children [7], and low access to clean water [8] compromise the effect of anthelminthics alone as a suitable means to control or eliminate STH infections. Indeed, two systematic reviews have largely failed to confirm the beneficial effects of periodic deworming [9, 10]. Thus, the development of a multivalent pan-anthelminthic vaccine targeting all three major STH infections would be a desirable biotechnology to prevent parasite reinfection and advance efforts for the control and elimination of these diseases [11].To advance such a strategy, two major *N*. *americanus* hookworm vaccine antigens are undergoing clinical vaccine tests, but there is a need to simultaneously develop *A*. *lumbricoides* and *T*. *trichiura* candidate antigens as suitable vaccines to be integrated within the human hookworm vaccine development program [12].

With regard to the development of *A*. *lumbricoides* vaccine antigens, the genetically highly homologous pig parasite, *Ascaris suum*, is commonly used as a model to identify and evaluate vaccine candidates. *A*. *suum* and *A*. *lumbricoides* are morphologically, immunologically, and genetically very similar [13, 14] and might even be subspecies variants. Indeed, *A*. *suum* has been shown to be an important cause of human ascariasis [15]. Similar to its natural host, the pig, mice can be infected with *A*. *suum* eggs and larvae will be released into the mouse intestine from which they will migrate to the lungs. However, in mice, these larvae cannot return to the intestine to develop into adult *Ascaris* worms [16–18]. Nonetheless, the mouse model has proven to be a valuable tool in the identification and evaluation of vaccine candidates against *Ascaris* infections [19–21]. Indeed, mice infected with *A*. *suum* eggs produced significant protection against *A*. *suum* egg challenge as judged by the significant reduction in the number of larvae migrating to the lungs or livers [19, 20, 22] and also by reduced lung pathology [23]. Using serum from infected mice or rabbit, several immunodominant antigens have been identified including As16 [20], As14 [19], As24 [24], As37 [21] and As-Enol (enolase) [25], and protective immunity has been induced by immunization with recombinant proteins [19, 20, 26, 27] and with DNA [28].

As14 and As16 were the first two antigens previously identified by the Tsuji lab through immunoscreening of an *A*. *suum* cDNA library with serum from *Ascaris* infected rabbit [19, 20]. They share 47% amino acid sequence identity and similar localization (in larva and adult stages, as well as in excretory/excretory products) [11]. Intranasal immunization with *Escherichia coli* expressed recombinant As16 and As14 conjugated with the cholera toxin B subunit (CTB) produced significant protection against *A*. *suum* infective egg challenge in mice [19, 20]. In addition, rAs16 induced protection in a pig animal model [29], and mice fed with As16-transgenic rice mixed with CTB were also protected against *A*. *suum* infection [30]. As14 fused with CTB was also successfully expressed in transgenic rice, but there was no oral immunization and protection reported [31]. Notably though, without CTB as the adjuvant, neither As16 nor As14 were able to induce protective immunity in any model.

Here, we report the production of recombinant As16 and As14 in the yeast *Pichia pastoris*, a eukaryotic expression system with scalability and without the concern for endotoxin contamination as in *E*. *coli*-expressed proteins [32]. Mice immunized with yeast-expressed rAs16 mounted a Th2-biased immune response and showed significant protection in terms of lung larval reduction. However, the same immunization regime for rAs14 did not induce any protection in mice. The immunological mechanisms underlying rAs16-induced protection were evaluated compared to rAs14 that did not induce protection. The results in this study provide a feasible approach to developing a vaccine against ascariasis on the basis of the yeast-expressed rAs16 that can be produced at low cost and formulated with alum, an FDA-approved adjuvant for human use that induces the Th2 immune response necessary to achieve anti-*Ascaris* immunity.

## Materials and methods

### Ethics statement

All animal procedures were conducted in accordance with Baylor College of Medicine Institutional Animal Care and Use Committee (IACUC) approved protocol AN-6297 in compliance with the Animal Welfare Act, PHS Policy, and other Federal statutes and regulations relating to animals and experiments involving animals.

### The *A*. *suum* mouse challenge model and parasite

*A*. *suum* eggs were originally obtained from an adult female worm collected from an infected pig at a pig slaughter house near Belo Horizonte, Brazil, and maintained in 0.2 N H_2_SO_4_ until most of them had developed into the embryonated infective stage (50–250 days). The infective embryonated eggs were shipped to our lab in Houston and used to orally challenge BALB/c mice as previously described [17]. The *A*. *suum* larvae hatch in the mouse intestine, and then migrate to the liver and lungs. The number of larvae recovered from mouse lung tissue eight days post infection were used as a biomarker to evaluate vaccine efficacy [17]. Crude extracts of *A*. *suum* eggs and lung-stage larvae were prepared by homogenization and sonication, and the insoluble pellet was removed by centrifugation as previously described [33].

### Sequence analysis

Amino acid sequences were aligned using CLUSTAL W and prepared for display using BOXSHADE. The phylogenetic trees were generated for As16 and its homologues from different nematodes using Phylogeny.fr [34] (http://www.phylogeny.fr/index.cgi).

### Expression and purification of recombinant As16 and As14 in *P*. *pastoris*

DNA coding for As16 without its signal peptide was codon optimized for expression in yeast and synthesized by GenScript. The DNA coding for As14 without its signal peptide was PCR amplified with As14 specific primers from *A*. *suum* larvae cDNA reverse-transcribed from total larval RNA. As16 and As14 coding DNAs were subcloned into the yeast expression vector pPICZαA (ThermoFisher Scientific, Carlsbad). The correct sequences and reading frames of the recombinant plasmids were confirmed by double-stranded DNA sequencing using vector flanking primers, α-factor and 3’-AOX1. The recombinant As16 and As14 (rAs16 and rAs14) with a hexahistidine tag at its C-terminus were expressed in yeast stain *P*. *pastoris* X-33 under induction with 0.5% methanol for 48–72 hours and then purified by immobilized metal ion affinity chromatography (IMAC), as described previously [35]. The purity of the recombinant proteins was determined by SDS–PAGE. The protein concentration was measured using BCA (ThermoFisher Scientific, Waltham) and Endotoxin clearance was confirmed using the Charles River Endosafe-PTS system (Charles River, Houston).

### Immunization and challenge infection

Six-week old female BALB/c mice were purchased from Taconic and divided into four groups of 20 animals each. Two vaccine groups were immunized subcutaneously with 50 μg of rAs16 or rAs14 emulsified with the adjuvant Montanide ISA720 (Seppic, Paris, France) in a total volume of 100 μl (antigen/ISA720 = 30/70 v/v). Mice were boosted twice with the same dose on days 21 and 35. The control groups were injected with PBS or PBS+ISA720 using the same regimen. Two weeks after the final vaccination, 5 mice from each group were sacrificed and blood and splenocytes were harvested for immunological tests. The remaining 15 mice from each group were challenged with 2,500 *A*. *suum* embryonated eggs in a total volume of 100 μl, administered by oral gavage. Eight days after infection, all infected mice were sacrificed, lungs were harvested, and *A*. *suum* lung-stage larvae were collected using a Baermann apparatus, as previously described [17]. Reduction in larval burden was calculated in all groups and the results were compared between the vaccine groups and the PBS and adjuvant control groups.

To improve the protection induced by rAs16 and interpret the immunological mechanism underlying the As16-induced protective immunity, another vaccine trial was performed by formulating 25 μg of rAs16 with either 200 μg of Alhydrogel (Brenntag, Mülheim, Germany), 20 μg of MPLA (InvivoGen, San Diego), or 50 μl of AddaVax (50/50, v/v) (InvivoGen, San Diego), each administered subcutaneously in a total volume of 100 μl per mouse given using the immunization regimen described above. Control groups were given adjuvant only. As a positive control, one group of 20 mice was orally infected three times with 1,000 *A*. *suum* embryonated eggs. After these immunizations, all mice were challenged with 2,500 *A*. *suum* embryonated eggs.

### Antibody assay

Sera from all blood samples were isolated and frozen at -20°C. The sera samples were assayed for antigen-specific IgG isotypes (IgG1, IgG2a) by a modified indirect enzyme-linked immunosorbent assay (ELISA). Briefly, individual wells of Nunc-Immuno Maxisorp plates (Thermo Scientific, Waltham) were each coated with 100 μl of rAs16 (3.1 μg/ml) or rAs14 (0.39 μg/ml) in coating buffer (KPL, Milford) overnight at 4°C based on the pretested optimal signal/noise ratio. The coated plates were blocked overnight with 0.1% BSA in PBST (PBS +0.05% Tween-20), then incubated with diluted serum samples, starting at 1:200 in 0.1% BSA in PBST for 2 hours. Horseradish peroxidase (HRP)-conjugated goat anti-mouse IgG1 and IgG2a (Lifespan Biosciences, Seattle) were used as secondary antibodies (1:4,000 in PBST). Sure Blue TMB (KPL, Milford) was added as the substrate. The reaction was stopped by adding 100 μL of 1 M HCl. The absorbance was measured at 450 nm using a spectrophotometer (BioTek, Winooski).

### Western blot analysis

Samples including crude extracts of *A*. *suum* lung-stage larvae and eggs and the recombinant proteins were separated by SDS–PAGE, then transferred onto PVDF membrane (ThermoFisher, Waltham). After blocking with 5% (w/v) skim milk powder in PBST, the membrane was incubated with sera from mouse immunized with recombinant proteins or *A*. *suum* eggs. HRP-conjugated goat anti-mouse IgG (Invitrogen, Carlsbad) was used as a secondary antibody. The antibody recognized bands were developed by ECL (GE Healthcare, Chicago). Recombinant Tc24 protein, expressed in yeast [36], was used as a negative control.

### Cytokine analysis by Luminex technology

Spleens were obtained from mice two weeks after the third immunization and the splenocytes were disassociated using a 100 μm cell strainer. The cells were then suspended in complete RMPI medium containing 10% heat inactivated FBS and 1x pen/strep solution. After being centrifuged at 300 x g for 5 min, the cells were resuspended in 2 mL ACK lysis buffer (Thermo Scientific, Waltham) for 5 min. After centrifugation the splenocytes were resuspended in complete RPMI media containing 10% DMSO and stored in liquid nitrogen until use.

For the cytokine stimulation assay, splenocytes were thawed in a 37°C water bath and transferred to 5 mL pre-warmed complete RPMI. Cells were washed once to remove residual DMSO. Splenocytes were seeded in a 96-well U-bottom culture plate (Falcon, Corning) at 1x10^6^ cells per well in 250 μl medium and re-stimulated with either 25 μg/mL rAs14 or rAs16 at 37°C, 5% CO_2_ for 48 hours. Positive controls were stimulated with 20 ng/mL PMA and 1 μg/mL Ionomycin, and unstimulated negative control cultures were performed concurrently. After 48 hours the cells were pelleted by centrifugation at 300 x g for 5 min and the supernatants were collected for measuring cytokine production. The supernatant samples were tested for levels of IL-2, IL-4, IL-5, IL-10, IL-12(p70), GM-CSF, IFN-γ and TNF-α using a Bio-Plex Pro Mouse Th1/Th2 8-plex kit (Bio-Rad, Hercules). To save material and costs, and to increase the sensitivity of the experiment, the kit was used in combination with DA-Bead plates (Curiox Biosystems, Singapore) as previously described [37]. Samples were run on a Bio-Plex Magpix multiplex reader according to manufacturer's recommendations (Luminex, Austin). Raw Luminex data were analyzed using the Bio-Plex Manager 6.0 software and plotted in GraphPad Prism 6.0. The cutoffs of the cytokine standards were dependent on the lot number of the Bio-Rad kit. To remove individual baseline cytokine values, cytokine values from non-restimulated samples were subtracted from those associated with antigen restimulated samples.

### Statistical analysis

Statistical significance of differences between groups was determined using a Mann-Whitney test using Prism 6. In Fig 5c the groups receiving the antigen + adjuvant were compared to the associated adjuvant alone group, the A. suum egg group, and to the PBS group using a Fisher’s LSD test. Data was presented as means ± standard deviation. For the statistical analysis, *p <* 0.05 was considered to be statistically significant.

## Results

### Expression of recombinant As16 and As14 proteins in yeast and their recognition by sera from *A*. *suum* infected mice

**Fig 1:**
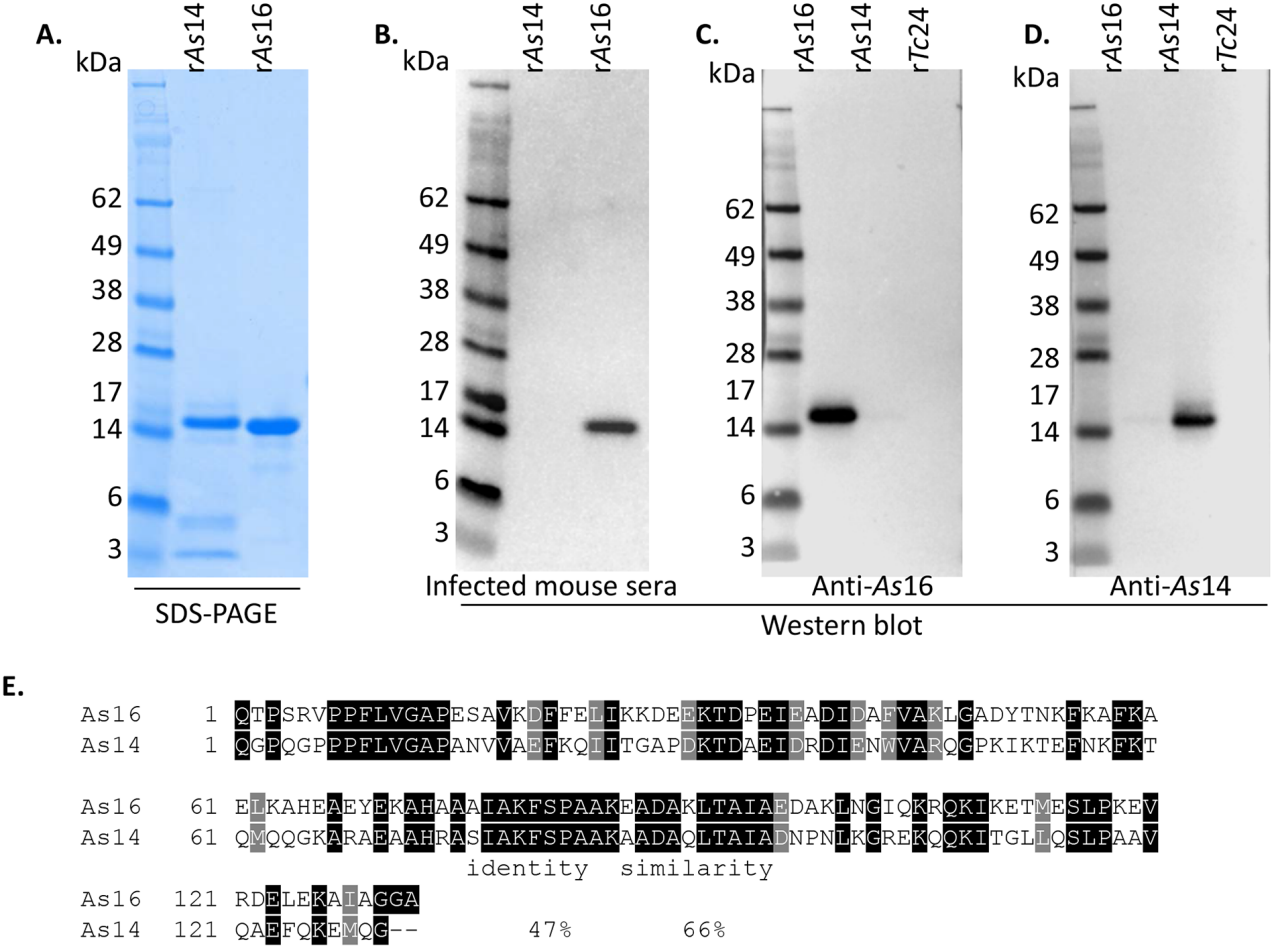
Properties of recombinant As14 and As16 proteins expressed in *P*. *pastoris* X-33. (**A**) SDS-PAGE of purified rAs14 and rAs16 proteins (1 μg each). (**B**) Western blot probed with sera from mice infected with *A*. *suum* eggs (diluted 1:3,000) shows that only rAs16 was recognized, but not rAs14 (all antigens 50 ng). (**C**) Western blot with anti-rAs16 mouse sera (1:3,000). (**D**) Western blot with anti-rAs14 mouse sera (1:3,000) (all antigens 50 ng). (**E**) Sequence comparison between As16 and As14 proteins shows 47% sequence identity and 66% similarity.

The genes encoding As16 (yeast codon optimized) and As14 (native sequence) without their signal peptides were cloned into the *Pichia* expression vector pPICZαA. The hexahistidine-tagged rAs16 and rAs14 proteins were expressed in *P*. *pastoris* X-33 through induction with 0.5% methanol over 72 hours and then purified by immobilized metal ion affinity chromatography (IMAC). The purified rAs14 and rAs16 proteins were analyzed by SDS–PAGE (Fig 1A). The apparent molecular weight for both proteins was approximately 15.0 kDa, which corresponds well to the sizes of the predicted gene products (14.8 kDa for rAs14 and 15.4 kDa for rAs16).

To determine whether rAs14 or rAs16 were recognized by protective immune sera from mice repeatedly infected with *A*. *suum* eggs described below (Protective immunity induced by immunization with rAs16), the infected mouse sera were used for Western blot. We observed that only rAs16 was recognized by the infected mouse sera, while rAs14 was not recognized by the same sera (Fig 1B). Even though the two proteins share 47% identity and 66% similarity in sequence (Fig 1E), there was no obvious immunological cross reaction between them when using rAs16 or rAs14 immunized mouse sera individually (Fig 1C and 1D). The results suggest that As16 antigen was exposed to the immune system during *A*. *suum* egg infection and larval migration, whereas As14 antigen may not have been immunologically accessible.

**Fig 2:**
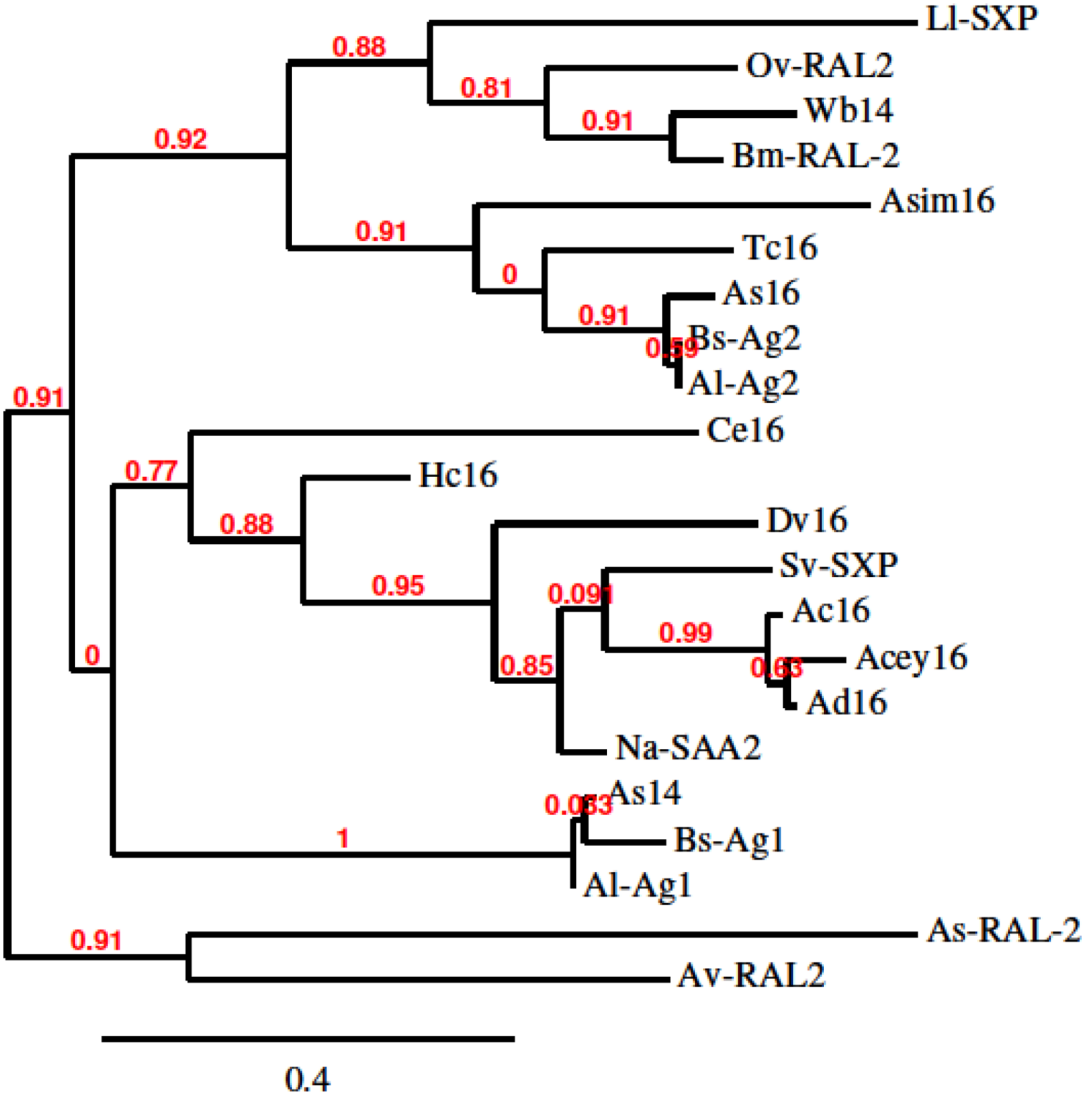
Phylogenetic tree of As16 and its homologues in other nematodes, with branch support values in red. The sequences include As16 (*A*. *suum*, GenBank accession# BAC66614.1); As14 (*A*. *suum*, BAB67769.1); Ce16 (*Caenorhabditis elegans*, NP_495640.1); Dv16 (*Dictyocaulus viviparus*, KJH51207.1); Acey16 (*Ancylostoma ceylanicum*, EPB72254.1); Ac16 (*A*. *caninum*, ABD98404.1); Ad16 (*A*. *duodenale*, KIH68079.1); Sv-SXP (*Strongylus vulgaris*, AGF90534.1); Na-SAA2 (*Necator americanus*, XP_013290850.1); Al-Ag1 (A. *lumbricoides*, ACJ03764.1); Bs-Ag1 (*Baylisascaris schroederi*, ACJ03761.1); Av-RAL2 (*Acanthocheilonema viteae*, AAB53809.1); Ll-SXP (*Loa loa*, XP_003142836.1); Ov-RAL2 (*Onchocerca volvulus*, P36991.1); WB14 (*Wuchereria bancrofti*, AAC17637.1); Bm-RAL-2 (*Brugia malayi*, XP_001900036.1); Bs-Ag2 (*B*. *schroederi*, ACJ03762.1); Al-Ag2 (*A*. *lumbricoides*, ADB45852.1); Hc16 (*Haemonchus contortus*, CDJ91573.1); Asim16 (*Anisakis simplex*, BAF43534) Tc16 (*Toxocara canis*, KHN84076.1) and As-RAL-2 (*Anisakis simplex*, BAF75709.1).

As16 is a 16 kDa nematode specific protein found among several different nematode species (Fig 2). It is present in different developmental stages of *A*. *suum*, including larvae and adult worms, but its function remains unknown [20]. As16 is highly conserved in *Ascaris spp*.; it shares 94% sequence identity with its counterpart in the human parasite *A*. *lumbricoides* (Al-Ag2), suggesting the possibility of achieving cross-protection for both *Ascaris* species if As16 were to be used as a vaccine antigen. Its homologues in filarial worms are ranked among the leading vaccine candidates against human onchocerciasis (*Ov*-RAL-2) [38] and *Brugia malayi* filarial infections (*Bm*-RAL-2) [39]. The As16 homologue from the canine hookworm (*Ancylostoma caninum*, *Ac*-16) also protected dogs from blood loss and reduced worm fecundity [40], and the *Baylisascaris schroederi* homologue, *Bs*-Ag2, protected giant pandas from infection with that parasite [41].

### Serological responses to immunization with rAs16 and rAs14 formulated with ISA720

**Fig 3:**
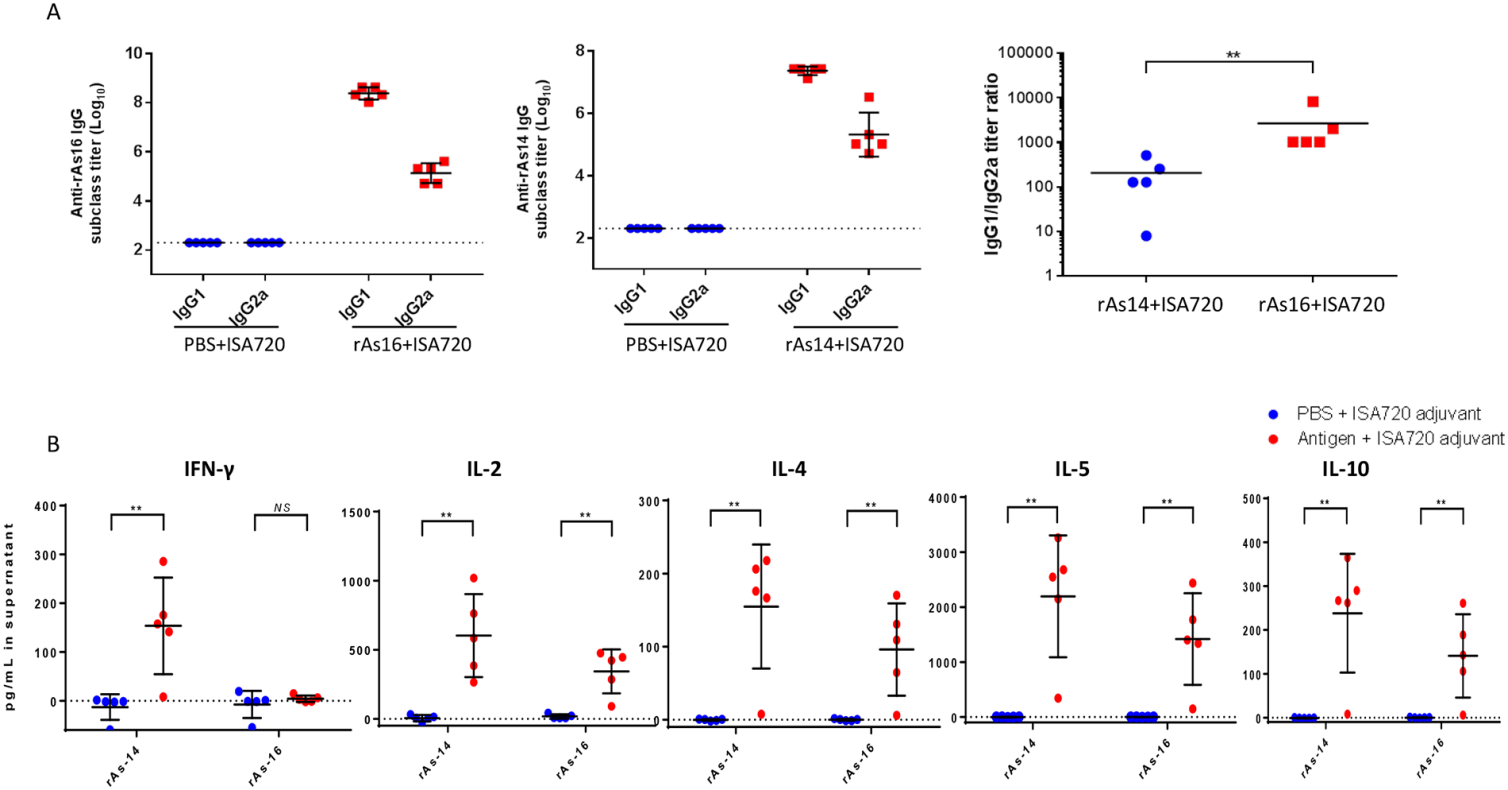
Mouse immune responses to the immunization of rAs16 and rAs14. **(A)** Anti-As16 and anti-As14 IgG1and IgG2a titers (Log10) in sera of BALB/c mice immunized with rAs16 and rAs14 formulated with ISA720, as measured by ELISA. The IgG1/IgG2a titer ratio for rAs16 immunization is higher than for the rAs14 immunization (2662:1 and 206:1, respectively). **(B)** Cytokine profiles (IFN-γ, IL-2, IL-4, IL-5 and IL-10) of BALB/c mice immunized with rAs16 and rAs14 formulated with ISA720. Cytokine levels were determined in supernatants of splenocytes after being re-stimulated with rAs16 or rAs14 (25 μg/ml) for 48 hours. Results are shown as means ± standard deviation (SD) and individual data points for each group (n = 5), **p<0.01, NS, non-significant.

Immunization with rAs16 and rAs14 formulated with the ISA720 adjuvant elicited significant titers of antigen-specific IgG1 and IgG2a antibodies in mice, with IgG1 as the predominant subclass (Fig 3A). The IgG1/IgG2a ratio after As16 immunization (2662:1) was more than 100-fold higher than the ratio for As14 (206:1) (Fig 3A), suggesting that a predominant Th2-type immune response occurs to both antigens, but in particular to rAs16. Mice given PBS+ISA720 did not show any IgG isotype responses specific to rAs16 or rAs14.

### Cytokine profiles of re-stimulated splenocytes from mice immunized with rAs16 and rAs14

To evaluate the cytokine profiles induced by immunization with rAs16 and rAs14, mice were sacrificed two weeks after the final immunization and their splenocytes were isolated. Cytokine profiles were determined by measuring levels of IL-2, IL-4, IL-5, IL-10 and IFN-γ in supernatants of splenocytes re-stimulated with 25 μg/ml rAs16 or rAs14 for 48 hours. Signal background in blank media was subtracted from re-stimulated samples. Statistically significantly increased levels of the cytokines IL-2, IL-4, IL-5, IL-10 were detected in the supernatants of re-stimulated splenocytes from mice immunized with rAs16 and rAs14; however, IFN-γ was significantly increased only in mice immunized with rAs14, not in the mice immunized with rAs16 (Fig 3B). Splenocytes from the PBS +ISA720 control group did not show any detectable cytokine expression.

### Protective immunity induced by immunization with rAs16, not with rAs14

**Fig 4:**
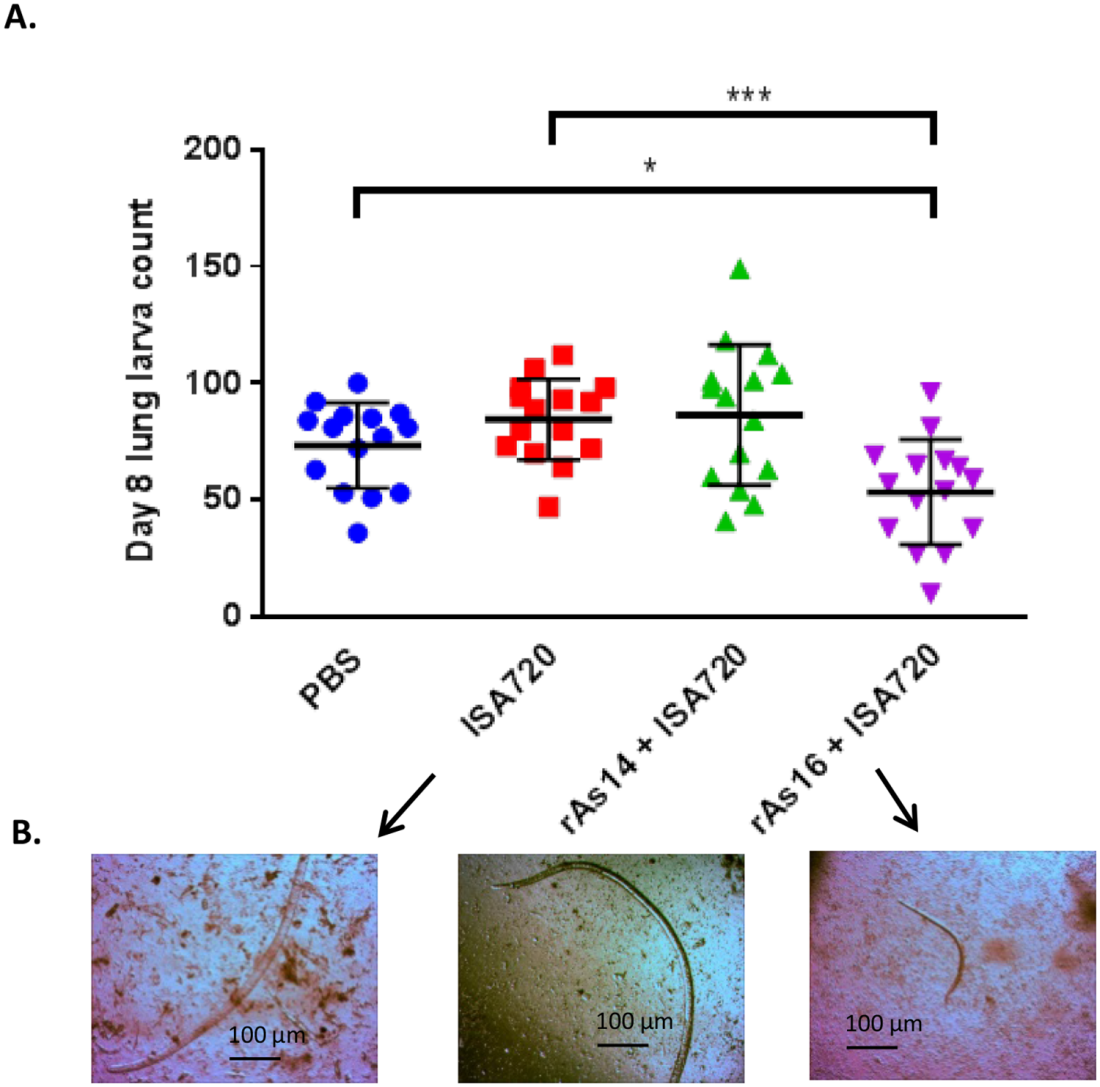
Lung larva reduction (A) and stunted development (B) in mice immunized with rAs16 formulated with ISA720 on Day 8 after being challenged with 2,500 *A*. *suum* eggs. Lung larvae are presented as the mean ± S.D (N = 15). The asterisks indicate statistically significant differences in larval reduction compared to the PBS or adjuvant control groups (*p< 0.05, ***p< 0.001). The larvae collected from lung Baermann culture were observed under 4x objective lens.

All mice were orally challenged with 2,500 *A*. *suum* embryonated eggs two weeks after the final immunization. *A*. *suum* larvae were collected from the lungs of immunized mice eight days after egg challenge. The lung larva count showed that mice immunized with 50 μg of rAs16 formulated with ISA720 adjuvant showed a 36.7% larva reduction compared to the adjuvant-only control groups, constituting a statistically significant difference (*p*<0.001) (Fig 4A). In addition, the size of the larvae collected from rAs16 immunized mice was much smaller than those collected from adjuvant control mice, suggesting developmental stunting due to the vaccination (Fig 4B). However, mice immunized with rAs14 did not show any protection in terms of reducing the number of larvae found in the lungs, or affecting the size of the larvae.

**Fig 5:**
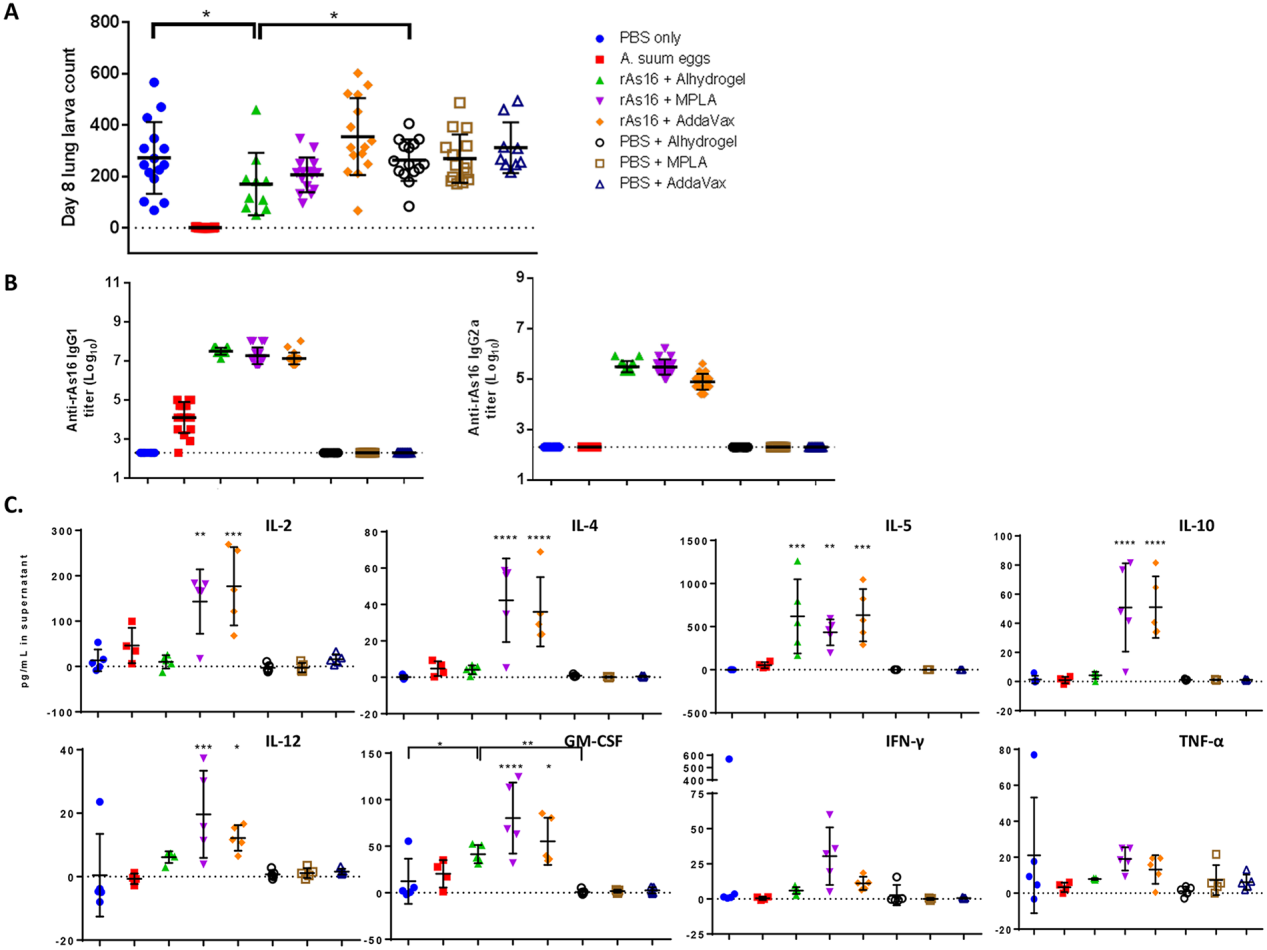
Protective immunity induced by immunization of mice with rAs16 formulated with different adjuvants (Alhydrogel, MPLA and Addavax). (**A**) Lung larval count on Day 8 after challenge with 2,500 *A*. *suum* eggs. Values are presented as the mean ± S.D. The asterisks indicate statistically significant differences (*p < 0.05) in lung larval reduction compared to the PBS and adjuvant control groups (n = 15). (**B**) Anti-As16 IgG1 and IgG2a titers (Log_10_) in sera from BALB/c mice immunized with rAs16 formulated with different adjuvants as measured by ELISA. Values are shown as means ± S.D and individual data points (n = 15). (**C**) Cytokine profiles (IL-2, IL-4, IL-5 and IL-10, IL-12, GM-CSF, IFN-γ and TNF-α) of BALB/c mice immunized with rAs16 formulated with different adjuvants. Cytokines detected in supernatants of splenocytes after stimulation with rAs16 (25 μg/ml) for 48 hours. Data are presented as means ± S.D and individual values for each group (n = 5). *p<0.05, **p<0.01 ***p<0.001, ****p<0.0001.

To further understand the immunological mechanism underlying the protective immunity induced by rAs16, and to select an adjuvant that performs best in protecting mice from infection, mice were immunized with only half the amount of As16 (25 μg, compared to 50 μg of rAs16 used for formulation with ISA720), formulated with three different adjuvants (Alhydrogel, MPLA and AddaVax). As a positive control, another group of mice was immunized through three trickle infections with 1,000 *A*. *suum* eggs. After three immunizations, all mice were challenged with 2,500 *A*. *suum* embryonated eggs. Mice immunized with 25 μg of rAs16 formulated with Alhydrogel, an alum adjuvant inducing a predominant Th2-type response, experienced a 38.9% lung larval reduction, which is statistically significant compared to the PBS and adjuvant-only control groups. However, rAs16 formulated with MPLA, a TLR4 agonist inducing a Th1/Th2-mixed type immune response, induced only a 26.1% lung larval reduction that was not statistically different from the control groups. Mice immunized with rAs16 formulated with AddaVax, an oil-in-water based adjuvant similar to the MF59 adjuvant that is licensed for flu vaccines in Europe and known to also induce a Th1/Th2 mixed immune response, were not statistically significantly protected against the *A*. *suum* egg challenge. Strikingly, mice immunized with three trickle infections with 1,000 *A*. *suum* eggs produced almost sterile immunity (99.6% lung larval reduction) against *A*. *suum* challenge (Fig 5A).

Serological antibody measurement revealed that mice immunized with rAs16 formulated with three different adjuvants all produced high titers of IgG1 and IgG2a, with a bias towards IgG1 (IgG1/Ig2a = 120–202) (Fig 5B). Interestingly, mice repeatedly infected with a low-dose of *A*. *suum* eggs produced a 99.6% lung larva reduction and also showed a significant increase of anti-As16 specific IgG1 antibodies, but without any accompanying IgG2a response. This suggests native As16 is released and exposed to the host immune system during repeated low-dose *A*. *suum* infections and may be involved in the induction of the observed Th2 protective immunity. Cytokines released by splenocytes upon re-stimulation of rAs16 showed that immunization with rAs16 formulated with Alhydrogel induced the release of IL-5, a major cytokine linked to Th2 responses in mice, and some level of IL-12 and GM-CSF. There were no detectable levels of IL-2, IL-4, IL-10, IFN-γ or TNF-α observed in mice immunized with rAs16 + Alhydrogel. Conversely, mice immunized with rAs16 formulated with MPLA and AddaVax showed a significant release of Th1 associated cytokines (IL-12, IFN-γ and TNF-α), Th2 type cytokines (IL-4, IL-5), and IL-10, IL-2 and GM-CSF as well (Fig 5C), even though there was no significant protective immunity observed in these Th1/Th2 adjuvant groups.

### Native As14 and As16 detected in lung larval stages of *A*. *suum*

**Fig 6:**
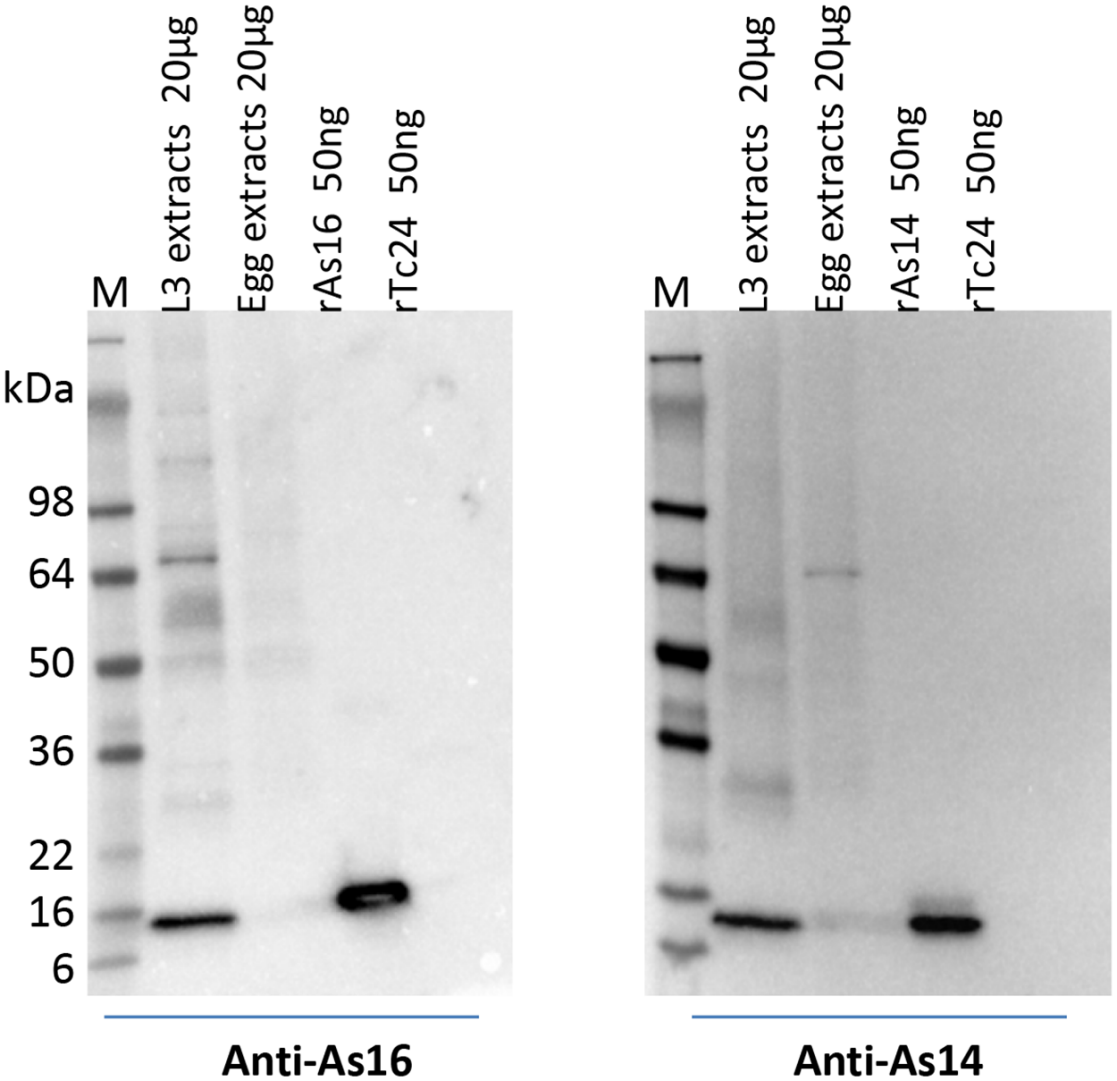
Native As16 and As14 expressed at the lung larval stage of *A*. *suum*. Western blots with anti-As16 or anti-As14 mouse serum (1:3,000) demonstrate that native As16 and As14 are expressed in L3 larvae collected from the lungs of infected mice, but not in infective eggs of *A*. *suum*. The unrelated *T*. *cruzi* Tc24 protein (50 ng) was used as a negative control.

Western blot analysis with sera from mice immunized with rAs16 and rAs14 demonstrated that anti-As16 mouse sera recognized a band at ~14 kDa in the soluble extracts of lung larvae of *A*. *suum*, but not in the extracts of *A*. *suum* eggs. For As14, mouse anti-As14 sera specifically recognized a band of about 13 kDa in lung larval extracts, but not in *A*. *suum* eggs, indicating native As16 and As14 are expressed only at the larval stage, not in the eggs of *A*. *suum* (Fig 6). As16 and As14 are likely expressed in *A*. *suum* larvae after hatching and during the migration to the lungs. However, since infected mice only produced antibodies to As16 but not to As14 (Fig 1), it appears that only As16 is exposed to the immune system during larval migration.

## Discussion

*A*. *suum* larval stage antigens induce significant protection through the reduction of worm larvae migrating to the lungs and thus subsequently reduce lung pathology [17, 22]. It was confirmed in this study that almost sterile protection with up to 99.6% lung larval reduction upon *A*. *suum* egg challenge was observed in mice infected three times with 1,000 *A*. *suum* eggs, indicating *A*. *suum* larvae produce some antigens that induce protective immunity during the migration of the L3 larvae to the lungs. Therefore, these larval antigens recognized by the immune serum from low-dose *Ascaris* egg-infected mice constitute potential vaccine antigen candidates. In previous studies, As16 and As14 had been identified as two such possible targets; immunization with the *E*. *coli*-expressed recombinant rAs16 or rAs14 proteins induced protective immunity in mice when adjuvanted with the cholera toxin B subunit (CTB) and administered through the intranasal route [19, 20, 29]. Indeed, in this study we identified that the protective immune sera from mice infected with *A*. *suum* eggs strongly recognized rAs16, confirming that As16 is an antigen which exposes to the host immune system and is immunogenic during *A*. *suum* infection, and the immune response to As16 may contribute to the protective immunity. Simultaneously, we confirmed that As16 is expressed in larvae, not in eggs. However, we did not observe that As14 was recognized by the *A*. *suum*-infected mouse immune sera and accordingly the immunization of As14 didn’t elicit any protective immunity against *A*. *suum* infection in this study, indicating that exposure of antigen is required for inducing protective immunity.

To develop a vaccine that can be used cost-effectively in endemic areas, we expressed As16 and As14 as recombinant proteins in the yeast *P*. *pastoris* X-33, a process that can be easily scaled up and manufactured without concerns for endotoxin contamination from proteins expressed in *E*. *coli* [32]. The purified rAs16 and rAs14 were used to test their vaccine efficacy in mice through subcutaneous administration when formulated with adjuvant ISA720. We found that mice immunized with yeast-expressed rAs16 produced a 36.7% lung larva reduction which is statistically significant compared to the adjuvant-only control. This level of protection was reproducible in another experiment with rAs16 formulated with Alhydrogel. In addition to the reduced number of larvae migrating to the lung, we also observed developmental stunting in those larvae that migrated to the lungs, suggesting immune responses induced by rAs16 damage the larvae’s viability and/or impair the development of survived larvae. We notice that As16 formulated with ISA720 and alum induced less lung larval reduction (36.7–38.9%) in this study compared to previous 58% induced by intranasal administration of As16 conjugated CBT as adjuvant in the previous study [20]. It is possibly due to the different adjuvants, immunization route or the post-translational modification of rAs16 expressed in *E*. *coli* and *P*. *pastoris* system that may induce different immune responses. The intranasal immunization may induce mucosal immunity in respiratory ducts that may contribute to the better protection against larva migrated to lungs [20], but there is no evidence that intranasal immunization could induce mucosal immunity in the gut in which the adult *Ascaris* worms parasitize. CTB is a nontoxic portion of cholera toxin, a toxin that causes massive watery diarrhea. CTB has the capacity not only to bind to monosialotetrahexosylganglioside on epithelia or antigen presenting cells to induce immune response as an adjuvant, but also to evoke a regulatory response that causes safety concern [42]. Therefore CTB is not widely used as an adjuvant for a human vaccine test. The effect of CBT as an adjuvant on inducing intestinal immunity against gastrointestinal pathogens through oral immunization is not well determined. The mice fed with rice transgenic with As16-CTB fusion were not able to induce enough immune response against *A*. *suum* infection unless CT was added [30]. Here we report mice subcutaneously immunized with As16 formulated with ISA720 and alum. The latter is a commonly used adjuvant approved by FDA for human use at low cost, and produced significant larval reduction in lungs upon *A*. *suum* infection, making As16 feasible and practical to be used for human trial.

The mechanism of protection induced by rAs16 remains under investigation. Possibly *A*. *suum* larva-secreted As16 is critical for the survival and development of the *A*. *suum* larvae in mammalian hosts. Therefore, the neutralization of this antigen through specific antibodies or other components of the immune responses may weaken the viability of the migrating larvae and block them from reaching the lungs, a critical step in migration back to intestine to develop to adult worms [22]. It has been demonstrated that anti-As16 antibodies inhibited the molting and survival of infective L3 when co-incubated together *in vitro* [29]. More specifically, immune-mediated protection appears to be associated with high levels of antigen-specific IgG1 and Th1/2 type cytokine secretion including elevated IL-4, IL-10 and INF-γ in previous studies [20, 29]. We observed that immunization with rAs16 formulated with ISA720 induced much higher antibody titers of IgG1 than IgG2a (2662:1) and elevated IL-4 and IL-5 but without any detectable IFN-γ response, indicating that the protection induced by immunization with rAs16 formulated with ISA720 in this study is associated with predominant Th2-type response rather than Th1-type response. IL-2 and IL-10 were also observed to be induced upon the immunization of As16 formulated with ISA720. Even though IL-2 is considered a Th1-biased cytokine, it also plays a central role in Th2 differentiation [43]. IL-10 has been essentially re-classified as a Treg-associated cytokine [44], but it is not clear if IL-10 is involved in the protective immunity since IL-10 was not induced in mice immunized with As16 formulated with Alhydrogel that produced similar protection. The protection of mice immunized with As16 formulated with Alhydrogel was associated with high level of IgG1 and IL-5, further confirming the Th2-type immune responses contribute mainly to the protective immunity of As16 against *A*. *suum* infection, This finding is consistent with other studies showing that a Th2-type response is critical in achieving protective immunity against helminthic infections [45–47].

As the second vaccine antigen candidate investigated in this study, As14 shares 47% sequence identical with As16 and produced protection in immunized mice when formulated with CTB in our previous study [19], we did not see any protection with immunization with As14 formulated with ISA720 adjuvant in this study. We did confirm expression of the antigen in *A*. *suum* larvae as well as strong immunogenicity when immunized in mice, but As14 was not recognized by immune sera from *A*. *suum* infected mice. Our findings suggest that As14 might not be exposed to the host immune system during infection so that immune response could not access it, or it might trigger a Th1-type immune response characterized with high level of IFN-γ that is not related to protection. Another possibility for not seeing protection with As14 immunization plus ISA720 in this study is that As14 may only induce protection when formulated with CBT and administered through the intranasal route that induces mucosal immunization [19].

Immunization with rAs16 alone, without an adjuvant, is not sufficient to yield protective immunity [20, 29], indicating that an adjuvant is necessary for boosting the rAs16-associated immune response or inducing a certain type of immune response that relates to protection. Since CBT may not be a suitable adjuvant for use in humans, we have tried some other adjuvants that are used commonly in vaccine efficacy tests. ISA720 is a water-in-oil emulsion that has been used in clinical trials for malaria vaccines and other infections [48, 49]. In this study, immunization with rAs16 emulsified with ISA720 induced a strong Th2-type immune response that was associated with lung larva reduction in immunized mice. To further interpret the protective immunological mechanism underlying the protection induced by As16, three other adjuvants (Alhydrogel, MPLA and AddaVax) were formulated with rAs16. Alhydrogel, an alum-based adjuvant approved by the FDA for use in humans, is known to stimulate a Th2-type response [50, 51]. MPLA (Monophosphoryl Lipid A) is a potent activator of TLR4 that boosts a combined Th1/Th2 response [52]. AddaVax is a squalene-based oil-in-water nano-emulsion adjuvant similar to MF59 that has been licensed in Europe for flu vaccines with the ability to induce a balanced Th1/Th2-type response [53, 54]. Our studies revealed that mice immunized with 25 μg of rAs16 formulated with Alhydrogel produced a similar level of lung larva reduction (38.9%) as observed with 50 μg of rAs16 formulated with ISA720 (36.7%); accompanied by high levels of IgG1 and IL-5, but without IFN-γ or TNF-α production. Immunization with rAs16 formulated with MPLA or AddaVax induced a mixed Th1/2-type responses, with high levels of both Th1 associated cytokines (IL-12, IFN-γ and TNF-α) and Th2 type cytokines (IL-4, IL-5, IL-10) and IL-2; however, this, at best, only resulted in a 26.1% larva reduction, which was not statistically different from the controls. Together, these results suggest that a Th1-type response might be counterproductive in terms of achieving protective immunity and might even interfere with the desired Th2-type response. Some evidence showed that IL-12 and IFNγ suppressed Th2 responses that caused chronic helminth and plasmodium co-infection [55]. In *Schistosoma* infections, Th1-type response did not contribute to protective immunity, but caused serious inflammatory pathology in the liver [56]. Other studies have found that the Th2-polarized protection induced by some nematode infections actually downregulated the Th1-type response [47, 57].

Interestingly, mice immunized with ISA720 and Alhydrogel formulated rAs16 that showed protection against *A*. *suum* egg challenge were all found to have high levels of IL-5. IL-5 plays a major role in the regulation of eosinophil formation, maturation, recruitment and survival [58]; eosinophils have been identified as important contributors in the control of helminth infections in mammalian hosts [59, 60]. IL-5 knockout mice, for instance, were incapable of augmenting blood and tissue eosinophil levels and were diminished in their ability to kill *Strongyloides stercoralis* larvae through innate and adaptive immune responses [61]. Thus, the induction of IL-5 in the immunized mice may recruit and activate eosinophils towards the migrating larvae and damage the parasite through antigen-dependent cellular cytotoxicity (ADCC) or through releasing toxic granules [59]. In addition to IL-5, granulocyte-macrophage colony-stimulating factor (GM-CSF) was also elevated in protected mice. In helminth infections, GM-CSF is known as a potent stimulator of granulocytes, namely eosinophils, basophils, and dendritic cells [62], all serving as first responders to the parasites [47]. It has been demonstrated that activated murine eosinophils can serve as specific antigen-presenting cells after infection with the cestode *Mesocestoides corti* [63], prime naïve T cells, and aid in the maturation of antigen-specific T-cells, for example upon *S*. *stercoralis* infection [64]. However, it cannot be concluded whether IL-5 or GM-CSF are involved in the protection since mice immunized with As16 formulated with MPLA and AddaVax also showed similar levels of IL-5 and GM-CSF but did not elicit significant protection. The real roles of IL-5 and GM-CSF in the As16 immunity may be more complicated and may associate with other factors.

We confirmed in this study that As16 is a good target for vaccine development against ascariasis. However, the As16-induced protection is not complete. Compared to the sterile immunity induced by *A*. *suum* infection, the partial protective immunity induced by As16 may reflect that As16 is just one of the larva-secreted protective antigens secreted by infected larvae, or trickling release of As16 from naturally infected larva may increase the immunogenicity and protection of As16. Non-sterile immunity or low protection rate is a common problem for vaccine development against helminth infections [65, 66], possibly due to the complexity of the life cycle of parasites, different antigens expressed by different stages, and immune-evasion strategies developed by helminths. However, the severity of helminth-caused diseases usually depends on worm burden, since low infection usually is asymptomatic [65]. Therefore, reducing the worm burden by vaccination, even not sterile, may benefit in reducing the seriousness of disease. To further increase the level of protection elicited by As16, its combination with other vaccine candidates such as As24 [26], As37 [21], and As-enolase [11, 25] is currently being investigated in a mouse model. The individual or combination of antigens will eventually be evaluated in a pig model, the permissive host of *A*. *suum* in which adult worms can be developed. As16 and other vaccine candidates are expected to be more effective at reducing worm burden in a pig model since most of these antigens are expressed on the both stages of larva and adult worm.

The ultimate goal is to develop a multivalent vaccine or vaccine combination with other STH vaccines such as Na-GST-1 and Na-APR-1, which are currently under clinical trial for preventing hookworm infection [12], to prevent infections or re-infections of more than one STH, as a complementary strategy for MDA to control STH endemic. The combination of different antigens from different STHs may increase protective immunity due to cross-protection since STHs are genetically related and share some sequence homology [11]. The individuals in endemic areas are usually repeatedly infected with *Ascaris* and other STHs. The existing immune responses to the infections may confound the vaccine efficacy; therefore the vaccine immunogenicity and efficacy should be tested in a re-infection animal model. Another concern for As16 as a surface-associated antigen of larval is IgE response during natural infection. Immunization of As16 and other larval antigens in individuals with existing IgE may induce allergic response as hookworm larva-secreted Na-ASP-2 does [67]. Therefore, immunoscreening for the IgE response in the endemic population is needed before the decision is made to use As16 for a vaccine trial in endemic area.

### GenBank accession numbers

As16 (*A*. *suum*, BAC66614.1); As14 (*A*. *suum*, BAB67769.1); Ce16 (*Caenorhabditis elegans*, NP_495640.1); Dv16 (*Dictyocaulus viviparus*, KJH51207.1); Acey16 (*Ancylostoma ceylanicum*, EPB72254.1); Ac16 (*A*. *caninum*, ABD98404.1); Ad16 (*A*. *duodenale*, KIH68079.1); Sv-SXP (*Strongylus vulgaris*, AGF90534.1); Na-SAA2 (*Necator americanus*, XP_013290850.1); Al-Ag1 (*A*. *lumbricoides*, ACJ03764.1); Bs-Ag1 (*Baylisascaris schroederi*, ACJ03761.1); Av-RAL2 (*Acanthocheilonema viteae*, AAB53809.1); Ll-SXP (*Loa loa*, XP_003142836.1); Ov-RAL2 (*Onchocerca volvulus*, P36991.1); WB14 (*Wuchereria bancrofti*, AAC17637.1); Bm-RAL-2 (*Brugia malayi*, XP_001900036.1); Bs-Ag2 (*B*. *schroederi*, ACJ03762.1); Al-Ag2 (*A*. *lumbricoides*, ADB45852.1); Hc16 (*Haemonchus contortus*, CDJ91573.1); Asim16 (*Anisakis simplex*, BAF43534), Tc16 (*Toxocara canis*, KHN84076.1) and As-RAL-2 (*Anisakis simplex*, BAF75709.1).
